# Chimeric switch and inverted cytokine receptors in T cell therapy: reprogramming T cells to overcome immune suppression in the solid tumor microenvironment

**DOI:** 10.3389/fimmu.2025.1662238

**Published:** 2025-10-08

**Authors:** Riley Rane, Fengqiao Li, Alexis Williams, Avaneesh Jayadev, Nhan L. Tran, Jeffrey A. Winkles, Gloria B. Kim

**Affiliations:** ^1^ Department of Physiology and Biomedical Engineering, Mayo Clinic Arizona, Scottsdale, AZ, United States; ^2^ Department of Immunology, Mayo Clinic Arizona, Scottsdale, AZ, United States; ^3^ Department of Cancer Biology, Mayo Clinic Arizona, Phoenix, AZ, United States; ^4^ Department of Neurological Surgery, Mayo Clinic Arizona, Phoenix, AZ, United States; ^5^ Department of Neurosurgery, University of Maryland School of Medicine, Baltimore, MD, United States; ^6^ Marlene and Stewart Greenebaum Comprehensive Cancer Center, University of Maryland, Baltimore, MD, United States

**Keywords:** solid cancers, CAR-T, immunotherapy, chimeric switch receptors, inverted cytokine receptors, immune suppression, synthetic biology

## Abstract

Adoptive T cell therapy has transformed cancer treatment, with chimeric antigen receptor (CAR) T cell therapy demonstrating remarkable clinical success in hematological malignancies. By genetically engineering a patient’s own T cells to recognize and attack cancer cells, CAR T therapy has achieved durable remissions in several blood cancers. However, its efficacy in solid tumors remains limited, largely due to the immunosuppressive tumor microenvironment (TME), which impairs T cell infiltration, persistence, and function. To address these challenges, innovative strategies are being developed to reprogram T cell signaling within the hostile TME. One promising class involves chimeric non-antigen receptors (CNARs), which modulate T cell activity independently of direct antigen recognition. Among these, chimeric switch receptors (CSRs) convert inhibitory checkpoint signals into activating cues, while inverted cytokine receptors (ICRs) redirect suppressive cytokine signals to promote T cell activation. In this review, we provide a focused overview of the design principles, mechanistic functions, and therapeutic potentials of CSRs and ICRs as adjuncts to CAR T therapy in solid tumors. We also discuss key considerations regarding safety, specificity, and clinical translation to inform future advancements in engineered receptor strategies for cancer immunotherapy.

## 1 Introduction

Adoptive T-lymphocyte therapy has emerged as a transformative approach for cancer treatment, offering a targeted strategy to harness the immune system against tumors (1, 2). Among its modalities, chimeric antigen receptor (CAR) T cell therapy has shown remarkable clinical success, particularly in hematological malignancies (3–6). However, its efficacy in solid tumors remains limited due to multiple challenges, including antigen heterogeneity, physical barriers, and most notably, the immunosuppressive tumor microenvironment (TME) that inhibits T cell activation and function (7–9). These hurdles underscore the urgent need for innovative strategies to enhance the specificity, persistence, and efficacy of CAR T cells within solid tumors.

**Figure 1:**
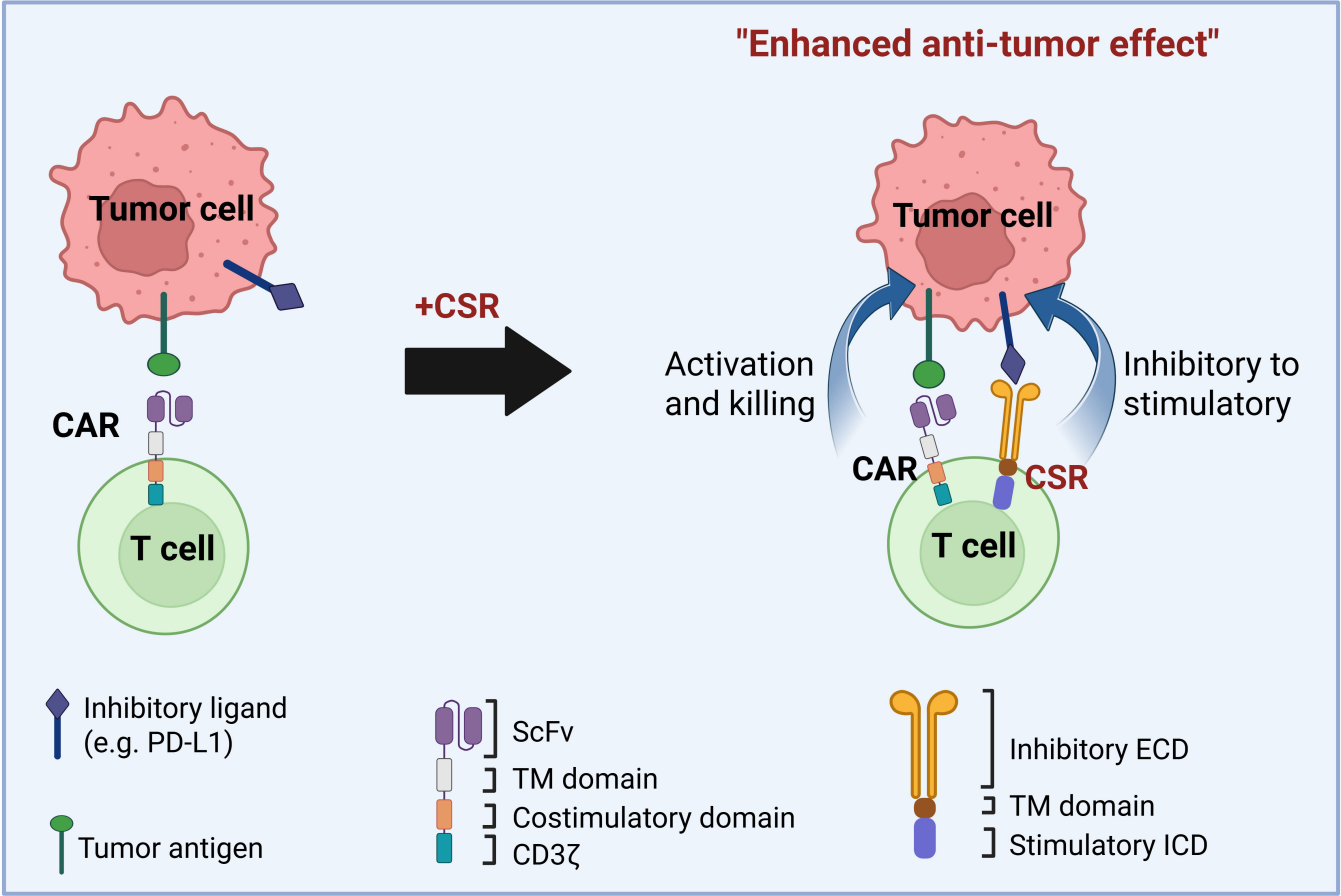
Design of chimeric switch receptors (CSRs) to enhance CAR T cell efficacy against solid tumors. CSRs are engineered receptors that bind to inhibitory ligands on tumor cells and convert these suppressive signals into stimulatory ones. CSRs consist of an extracellular inhibitory ligand-binding domain (ECD), a transmembrane (TM) domain, and a stimulatory intracellular signaling domain (ICD). In the figure, second-generation chimeric antigen receptors (CARs) contain a single-chain variable fragment (scFv) for antigen recognition, a hinge, a TM domain, a costimulatory domain, and a CD3ζ signaling domain. When co-expressed in CAR T cells, CSRs promote T cell activation, proliferation, and antitumor function by reversing inhibitory cues in the tumor microenvironment. CSRs can function independently or synergize with CARs or T cell receptors (TCRs) to enhance T cell responses against tumors.

One promising strategy involves chimeric switch receptors (CSRs), engineered receptors that convert inhibitory signals into activating ones within the TME (10). Under physiological conditions, immune checkpoint receptors such as programmed death-1 (PD-1) and cytotoxic T-lymphocyte-associated protein 4 (CTLA-4) serve as negative regulators of T cell activity by engaging ligands like PD-L1, thereby maintaining immune homeostasis and preventing autoimmunity (11, 12). Many solid tumors exploit these pathways by upregulating such ligands, leading to chronic inhibitory signaling, T cell exhaustion, and diminished anti-tumor responses (13–15). CSRs counteract this suppression by fusing the extracellular domains of inhibitory receptors with intracellular signaling domains of costimulatory molecules like cluster of differentiation 28 (CD28) and 4-1BB (CD137). This design enables T cells to transform immunosuppressive signals into costimulatory cues, thereby enhancing T cell activation and persistence in the hostile TME (**Figure 1**) (12, 16–18).

CSRs belong to a broader class of synthetic receptors termed chimeric non-antigen receptors (CNARs), which modulate T cell activity independently of direct antigen recognition (19). Within this class, various receptor designs have emerged to bolster T cell responses in immunosuppressive environments. One such subclass includes inverted cytokine receptors (ICRs), which similarly aim to overcome immunosuppressive cues. ICRs convert suppressive cytokine signals, such as those from transforming growth factor-β (TGF-β) (18, 20, 21) or granulocyte-macrophage colony-stimulating factor (GM-CSF) (22), into stimulatory outputs that promote T cell activation. However, unlike CSRs, which directly rewire inhibitory checkpoint pathways into stimulatory signals, ICRs rely on the local cytokine milieu and can exhibit context-dependent effects that vary across tumor types. Their inclusion here highlights the expanding landscape of receptor engineering strategies targeting immune suppression, though mechanically and functionally, ICRs represent a distinct and more variable approach compared to CSRs.

This review provides a focused overview of engineered receptor strategies designed to enhance T cell function in the TME, with a particular focus on CSRs and ICRs. While CSRs directly reprogram inhibitory checkpoint signals into costimulatory cues, ICRs harness immunosuppressive cytokines and redirect their signaling to promote T cell activation. Together, these complementary approaches exemplify the versatility of CNARs, a growing class of synthetic receptors modulating T cell behavior independently of direct tumor antigen recognition. Although still limited in number, a few early-phase clinical trials are exploring the safety and efficacy of CSR-engineered T cells, underscoring their emerging translational potential. By dissecting their mechanisms, design principles, and therapeutic potential, we highlight how CSRs and ICRs can be leveraged to overcome immune suppression and improve the efficacy of CAR T cell therapy in solid tumors.

## 2 Targeting the PD-1 pathway

### 2.1 PD-1 signaling pathway

PD-1 is a type 1 transmembrane protein that is widely expressed on immune cells, including B cells, tumor-associated macrophages (TAMs), and is most notably enriched on tumor-infiltrating T cells, where it plays a key role in suppressing antitumor immunity (23–25). Alongside CTLA-4, PD-1 functions as a key immune checkpoint receptor that inhibits T cell activity in the TME. These two checkpoint receptors have been extensively studied for their roles in tumor immune evasion, leading to the development of several FDA-approved PD-1 inhibitors for clinical use (26, 27). In addition to its role in cancer, PD-1 signaling is critical for maintaining immune homeostasis and self-tolerance by preventing excessive immune activation. This regulation helps protect against autoimmune diseases, chronic inflammation, and T cell exhaustion under physiological conditions (28–30). Among its known ligands, programmed death ligand-1 (PD-L1) is the primary binding partner of PD-1 and is frequently upregulated on both tumor cells and immunosuppressive stromal cells. Upon binding to PD-L1, PD-1 inhibits T cell receptor (TCR) signaling (31) by recruiting the phosphatases SHP-1 and SHP-2, which dephosphorylate key signaling molecules such as PI3K/AKT and ZAP70. This results in reduced cytokine production, T cell proliferation, and cell cycle progression (32). Additionally, PD-1/PD-L1 interactions indirectly suppress TCR signaling by inhibiting the activity of casein kinase 2 (CK2), further dampening T cell responses (23).

Beyond modulating T cell activation, recent studies have shown that PD-1/PD-L1 signaling also contributes to other aspects of immune regulation, including dendritic cell migration. A study by Kantheti et al. (33) demonstrated that PD-1/PD-L1 interactions influence the trafficking of DCs, suggesting that this pathway plays a broader role in coordinating immune responses across multiple cell types.

In cancer, the PD-1/PD-L1 pathway is exploited by tumor cells to evade immune surveillance. Tumor and stromal cells in the TME upregulate PD-L1 expression, thereby enhancing PD-1-mediated inhibitory signaling and suppressing anti-tumor immune responses (34, 35). In cases of adaptive immune resistance, tumors take advantage of the natural physiology of PD-L1 induction, particularly the secretion of proinflammatory cytokines, to activate PD-1 signaling (24). These cytokines, particularly IFN-γ and TNF-α, are secreted by activated tumor infiltrating lymphocytes (TILs) (10). Sustained exposure to high levels of PD-L1 in the TME leads to persistent upregulation of PD-1 on TILs, driving T cell exhaustion and impairing effective immune surveillance (36). Moreover, PD-1/PD-L1 signaling facilitates the recruitment and maintenance of regulatory T cells (Tregs) and other immunosuppressive cell types, further reinforcing a tolerogenic microenvironment that supports tumor growth and immune escape (37).

### 2.2 PD-1-based CSRs

**Figure 2:**
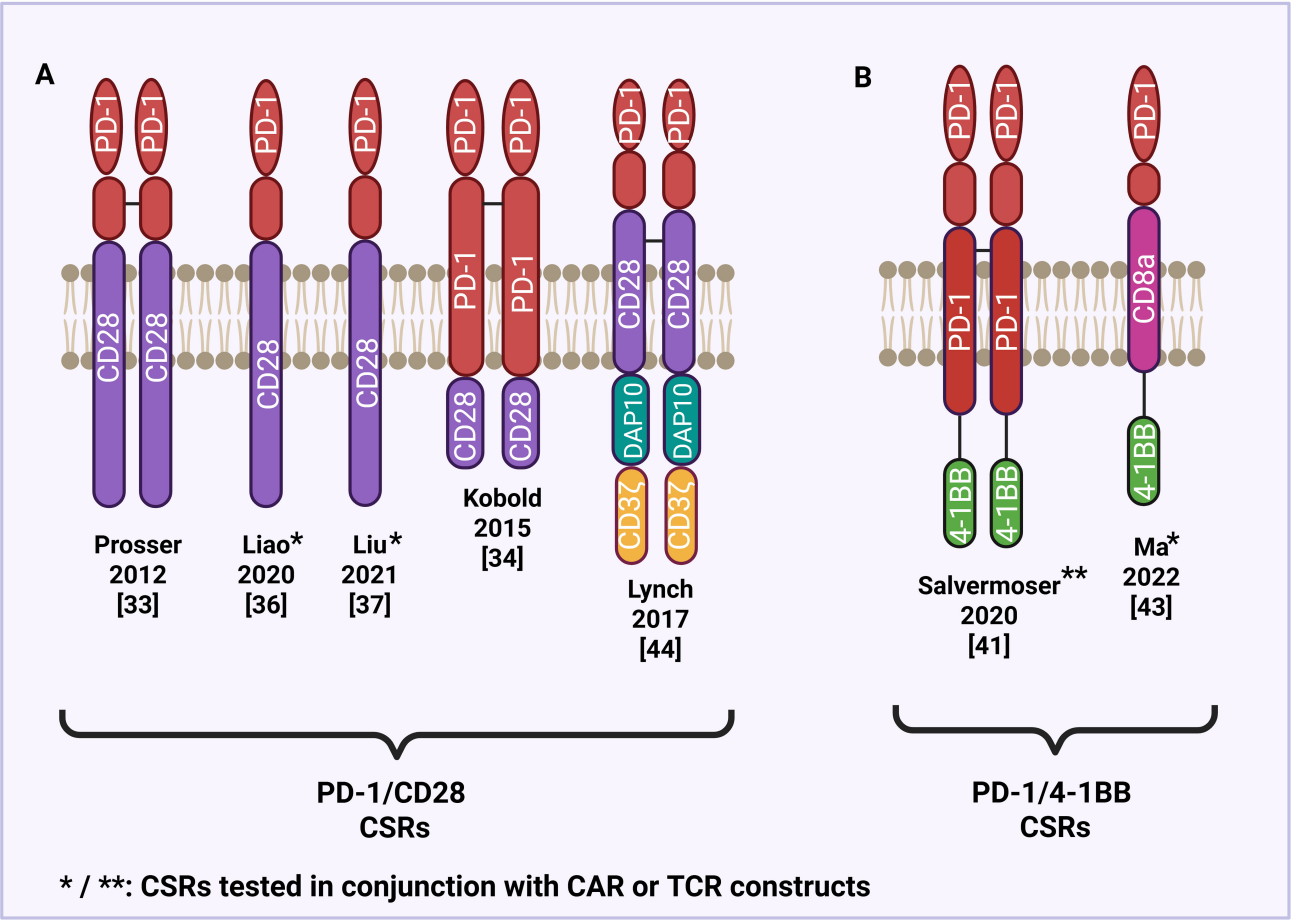
Structural design of PD-1-based CSRs. **(A)** PD-1/CD28 CSRs with a CD28 transmembrane and/or intracellular domain. **(B)** PD-1/4-1BB CSRs with a 4-1BB intracellular domain.

One of the most well-characterized CSRs combines the extracellular domain of PD-1 with the intracellular signaling domain of CD28, a key costimulatory receptor that promotes T cell activation (**Figure 2A**). Prosser et al. (38) initially developed PD-1/CD28 CSRs by substituting the transmembrane and intracellular domains of PD-1 with those of CD28 and transduced them into CD8+ cytotoxic T lymphocytes. These engineered T cells retained PD-L1 binding while exhibiting enhanced ERK phosphorylation, cytokine production, and proliferative capability. While these early constructs incorporated both the transmembrane and intracellular domains of CD28, more recent studies have focused on hybrid receptors that retain PD-1 transmembrane domain and fuse only the intracellular domain of CD28. Kobold et al. (39) demonstrated that this conformation significantly improved T cell activation, cytokine release, and tumor cell killing, likely due to increased CSR surface expression and greater binding affinity for the PD-L1 ligand.

Recent clinical studies have evaluated the safety and bioactivity of PD-1/CD28 CSRs in patients (**Table 1**). In a Phase I Clinical Trial (NCT02937844), Guo et al. (40) treated PD-L1-positive glioblastoma patients with PD-1/CD28-engineered T cells. Treatment led to increased T cell infiltration and elevated levels of IFN-γ and IL-6 in cerebrospinal fluid, with no adverse events beyond grade 2, neurotoxicity, or cytokine release syndrome (CRS), indicating the safety profile of CSRs. Although this clinical trial involved a limited number of patients, another clinical trial (NCT02930967) has also evaluated the safety and efficacy of PD-1 CSRs in recurrent or metastatic malignancies.

**Table 1:** Clinical studies of chimeric switch receptor-mediated treatment.

Strategy	Cell Product	Engineering	Indication	NCT identifier	Status
CSR	Anti-PD-L1 CSR T cells	Anti-PD-L1 CSR	Recurrent Glioblastoma Multiforme	NCT02937844	Phase 1 (40)
	Anti-PD-L1 CSR T cells	Anti-PD-L1 CSR	Recurrent PD-L1+ Malignant Tumors; Metastatic PD-L1+ Malignant Tumors	NCT02930967	Phase 1

While CSRs offer a powerful approach by converting inhibitory signals into activating ones, genetic knockout (KO) strategies – such as CRISPR/Cas9-mediated deletion of inhibitory checkpoint receptors like PD-1-represent an alternative that removes inhibitory signaling altogether. PD-1 KO T cells have shown enhanced motility, effector functions, and improved *in vivo* performance in preclinical infection and tumor models (41, 42). However, knockouts may also disturb regulatory or homeostatic functions of the receptor, potentially leading to dysregulation or exhaustion impacts (43, 44). In comparison, CSR designs can preserve some of the receptor’s regulatory architecture by replacing or repurposing cytoplasmic signaling domains, perhaps mitigating risks associated with total loss of function.

### 2.3 PD-1-based CSRs combined with CARs

Inhibitory signaling pathways, such as PD-1/PD-L1 interactions, not only suppress immune function in natural T cells but also limit the therapeutic efficacy of CAR T cells in cancer. To address this, recent studies have explored combining PD-1 CSRs and CAR constructs to enhance CAR T cell performance. For example, Liao et al. (45) developed a first-generation dual-targeting CD19/HER2 CAR, co-expressed with a universal PD-1/CD28 CSR, and evaluated its functionality and antitumor efficacy both *in vitro* and *in vivo* using human tumor xenograft mouse models. The engineered CAR T cells displayed comparable cytotoxic activity against CD19/HER2+ tumor cells regardless of PD-L1 presence but showed increased proliferation and cytokine release in the presence of PD-L1. Importantly, in the absence of the tumor antigens CD19 and HER2, these CAR T cells exhibited no cytotoxicity against PD-L1^+^ cells, indicating safety for normal tissues while maintaining the ability to eliminate tumor cells with low tumor antigen expression. These results suggest that PD-1 CSRs can both minimize on-target, off-tumor toxicity and function as “immune accelerators” by counteracting PD-L1-mediated inhibition to enhance tumor-lytic activity of engineered T cells. In one of the first in-human studies of CSR/CAR T therapy (NCT03258047), Liu et al. (46) evaluated CD19-targeting CAR T cells co-expressing PD-1/CD28 CSRs in patients with PD-L1^+^ B cell lymphoma. Compared to conventional anti-CD19 CAR T cells, the CD19-PD-1/CD28 CAR T cells exhibited superior antitumor activity, with enhanced T cell proliferation, cytokine production, and cytotoxicity observed both *in vitro* and *in vivo*. Clinically, these engineered T cells were well-tolerated, as patients experienced no severe neurologic toxicity or symptoms of CRS. While early-phase trials such as NCT04850560 and NCT03932955 are also evaluating the efficacy of CD19-targeting CAR T cells co-expressing a PD-1/CD28 CSR, larger clinical studies are still needed to fully assess their therapeutic potential (47, 48).

### 2.4 PD-1-based CSRs with other costimulatory domains

Aside from CD28, alternative costimulatory domains have been incorporated into CSRs to improve T cell activation and persistence. One such domain is 4-1BB a member of the tumor necrosis factor receptor (TNFR) superfamily, known to promote T cell survival and memory formation (**Figure 2B**) (49). Salvermoser and others (50, 51) investigated the efficacy of PD-1/4-1BB switch receptors in combination with preferentially expressed antigen in melanoma (PRAME)-specific TCRs to enhance T cell function under chronic antigen stimulation. Using both 2D and 3D *in vitro* models that mimic immunosuppressive conditions, they found that PD-1/4-1BB CSR expressing T cells exhibit improved cytotoxicity, proliferation, and persistence in the presence of PD-L1, supporting the use of 4-1BB as a costimulatory domain capable of reversing PD-1-mediated inhibition. Ma et al. (52) further evaluated the efficacy of PD-1/4-1BB CSRs coexpressed with second-generation HER2-specific CAR T cells in treating pleural and peritoneal metastasis. The study found that the CSRs enhanced the functionality of the anti-HER2 CAR T cells in terminating metastatic tumors in xenograft mouse models and showed increased expression of T cell activation and proliferation. These preclinical findings led to the initiation of a Phase I clinical trial (NCT04684459) investigating PD-1/4-1BB CSRs in patients with pleural or peritoneal metastasis. Another promising costimulatory domain used in CSR design is DNAX-activating protein 10 (DAP10), which like CD28 and 4-1BB, promotes immune cell activation, but through a distinct signaling cascade. DAP10 has been shown to promote T cell effector function and induce signal transduction in a manner that favors therapeutic efficacy in immunosuppressive setting. Lynch et al. (53) sought to explore the therapeutic potential of PD-1/DAP10 CSRs when compared to PD-1/CD28 CSRs, specifically when treating lymphoma. PD-1/DAP10 was found to induce a central memory phenotype in murine effector CD8 T cells, leading to greater persistence and anti-tumor immunity *in vivo*. These findings suggest that DAP10-based CSRs may drive distinct cytokine profiles and T cell differentiation states, offering an alternative costimulatory platform with therapeutic advantages over traditional CD28-based constructs.

## 3 Targeting other signaling pathways

**Figure 3:**
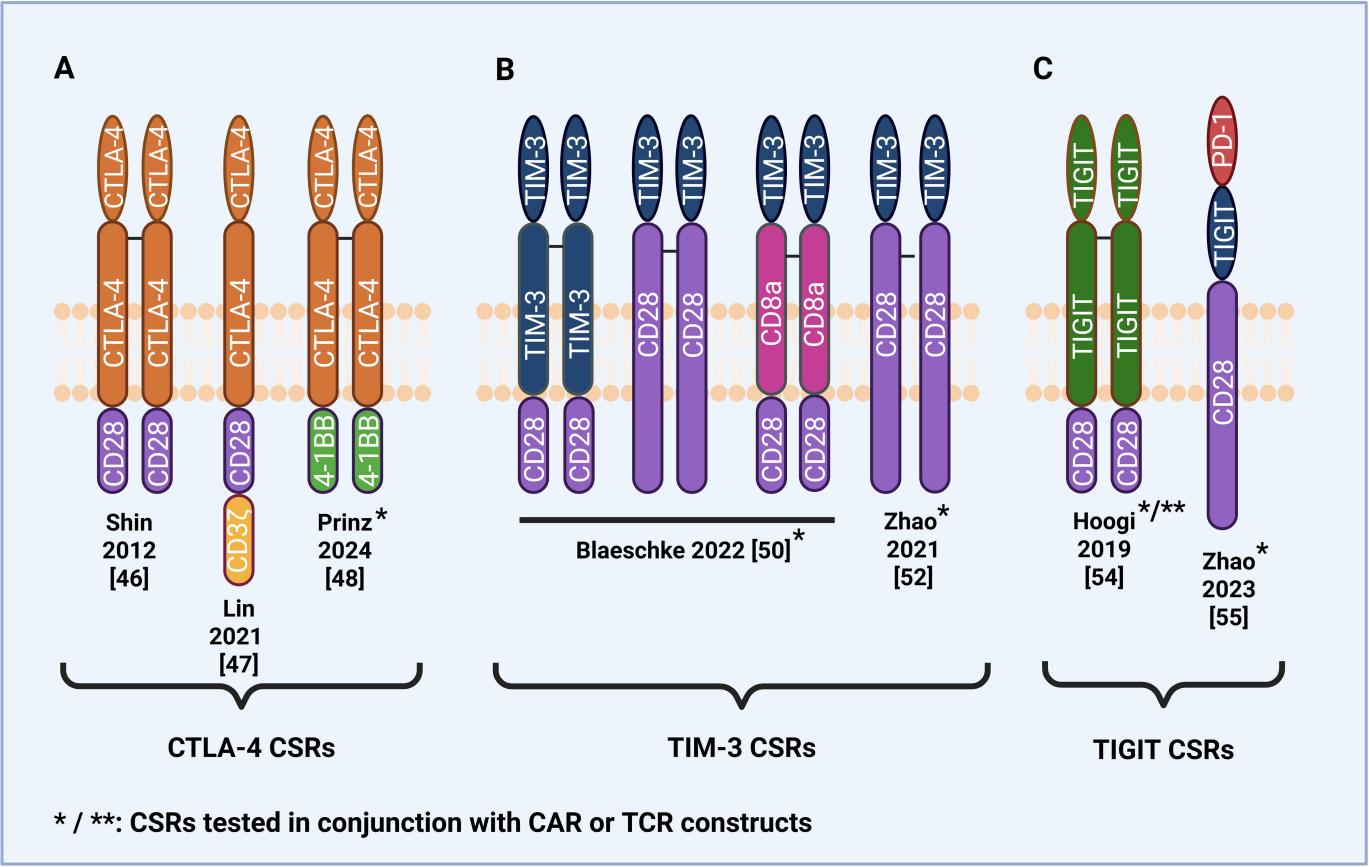
Structural design of CSRs and CAR/CSRs based on immune checkpoint receptors other than PD-1. **(A)** CTLA-4-based CSRs and CAR/CSRs with a CD28 or 4-1BB intracellular domain. **(B)** TIM-3-based CSRs and CAR/CSRs with a CD28 intracellular and/or transmembrane domain. **(C)** TIGIT-based CSR with a CD28 intracellular domain and dual PD-1/TIGIT/CD28 CAR/CSR.

Besides PD-1, several other inhibitory immune checkpoint receptors on T cells are exploited by tumors to evade immune surveillance. Recent studies have explored engineering CSRs from these inhibitory receptors, including CTLA-4, T cell immunoglobulin and mucin domain-containing protein 3 (TIM-3), and T cell immunoreceptor with Ig and ITIM domains (TIGIT), in order to convert inhibitory signals to stimulatory signals. This strategy aims to overcome immune suppression within the TME (**Figure 3**).

### 3.1 CTLA-4-based stimulatory switch receptors

CTLA-4 is type 1 transmembrane receptor expressed on T cells that functions as a negative regulator of immunity following T cell activation (10). As an inhibitory receptor, CTLA-4 plays a critical role in maintaining immune homeostasis along with stimulatory receptor CD28 by mediating a balance between stimulatory and inhibitory signals. In suppressing T cell activation, CTLA-4 also serves to prevent autoimmunity.

CTLA-4 transmits an inhibitory signal to activated T cells via two primary mechanisms: 1) cell-intrinsic inhibition: CTLA-4 recruits phosphatases such as SHP2 and PP2A to its cytoplasmic tail, leading to the dephosphorylation of key signaling molecules involved in T cell activation, including LAT and ERK (54); 2) cell-extrinsic inhibition: CTLA-4 competes with CD28 for binding to the B7 ligands CD80 and CD86, which are expressed on antigen-presenting cells (APCs) (10). CTLA-4 has a much higher binding affinity for these ligands than CD28, effectively sequestering them and blocking CD28-mediated recruitment of PI3K, Grb2, and Vav1, molecules critical for delivering the costimulatory signals required for T cell proliferation and function (10, 55).

Tumors can exploit CTLA-4-CD80/86 axis to evade immune surveillance by upregulating CD80/86 expression (56), thereby chronically engaging CTLA-4 and delivering sustained inhibitory signals that promote T cell exhaustion. To overcome this immunosuppressive mechanism, several CTLA-4-based CSRs have been developed by combining the extracellular and transmembrane domains of CTLA-4 with the intracellular domains of CD28 and 4-1BB (**Figure 3A**). Shin et al. (55) were among the first to introduce a CTLA-4/CD28 CSR into both CD8 and CD4 T cells and found it improved antitumor effects, including increased cytokine secretion of IFN-γ and IL-2, in murine tumor models. Lin et al. (56) developed a CTLA-4/CD28 CSR that exhibited enhanced antitumor activity against CD80/86-positive B cell malignancies. *In vitro*, CSR-expressing T cells secreted higher levels of IFN-γ and IL-2 and demonstrated higher cytotoxicity. Significant decreases in tumor volume and weight were also observed when the CSR-expressing T cells were tested *in vivo* in patient-derived xenograft mice models. However, regarding safety, the study found that CSR-expressing T cells exhibited toxicity against non-malignant CD80/86-positive cells, raising the concern of off-target effects. Another safety concern was that mice infused with CSR-expressing T cells were observed to experience mild graft-versus-host-disease (GvHD) and mild cytokine release syndrome (CRS), though neither proved lethal.

While the studies found that CTLA-4/CD28 CSRs enhanced anti-tumor efficacy, concerns about potential safety risks underscore the importance of developing CSRs with improved *in vivo* safety and tumor specificity. Prinz et al. (57) addressed this by designing a novel CAR/CSR construct consisting of a first-generation anti-CD19 CAR co-expressed with a CTLA-4/4-1BB CSR, intended to reduce off-target effects on healthy cells by selectively targeting cells expressing CD19 and CD80/CD86. The study demonstrated that CAR/CSR T cells exhibited enhanced cytotoxicity and increased secretion of IFN-γ and IL-2 compared to second-generation CAR T cells when co-cultured with Burkitt lymphoma cells overexpressing CD80/CD86. *In vivo*, CAR/CSR treatment resulted in higher complete remission rates in a first-line mouse model and significantly prolonged survival in a second-line model of tumor relapse, suggesting a promising strategy to enhance the efficacy of anti-CD19 CAR T cell therapy for relapsed/refractory B cell lymphoma. Regarding safety, the CAR/CSR construct induced reduced secretion of interleukin-6 (IL-6). This cytokine is commonly elevated during cytokine release syndrome (CRS), and its reduction indicates the potential to mitigate the risk or severity of CRS.

Finally, Park et al. (58) investigated CTLA-4/CD28 CSR in the context of allogeneic T cell therapies, where donor-derived T cells are used to treat patients. The study found that CSR expression enhanced the graft-versus-tumor (GVT) effect in models of relapsed hematologic malignancies, such as acute lymphoblastic leukemia (ALL). However, to mitigate the associated increase in GvHD risk, the study co-administered IL-10-overexpressing mesenchymal stem cells, providing an immunosuppressive buffer to preserve efficacy while improving safety.

### 3.2 TIM-3-based stimulatory switch receptors

TIM-3 is a type 1 transmembrane protein expressed on immune cells including activated T cells, NK cells, myeloid cells, and Treg cells (59). Upon T cell activation, TIM-3 is upregulated to maintain T cell homeostasis by inhibiting T cell mediated cytotoxicity (60). Unlike other checkpoint receptors, TIM-3 lacks any known inhibitory signaling motifs in its intracellular domain, but contains conserved tyrosine residues that may mediate alternative signaling functions (59). Ligand binding (e.g. galectin-9, high mobility group box protein 1 (HMGB1), carcinoembryonic antigen cell adhesion molecule 1 (CEACAM1), and phosphatidylserine) induces phosphorylation of these tyrosines, leading to dissociation of BAT3 and enabling TIM-3-mediated inhibition of T cell responses (59, 60). As with other checkpoint receptors, tumor cells exploit this pathway by upregulating TIM-3 ligands to suppress anti-tumor immunity.

TIM-3 ligands are expressed on the surface of almost all tumor types, making them far more widely expressed compared with ligands of other checkpoint receptors PD-1, CTLA-4, and TIGIT (60). Despite this widespread expression, relatively few TIM-3-based CSRs have been developed and tested (**Figure 3B**). Zhao et al. (61) were the first to develop a TIM-3/CD28 CSR by fusing the extracellular domain of TIM-3 with the transmembrane and intracellular domains of CD28 and test its efficacy *in vitro* and *in vivo*. The study found that the CSR-expressing anti-CD19 4-1BB CAR T cells exhibited enhanced cytotoxicity via the IL-21/Stat3 axis, increased cytokine secretion, and decreased exhaustive phenotype compared to second-generation 4-1BB-based anti-CD19 CAR T cells (61). Upon repeated infusions of CSR-expressing CAR T cells, there were remarkably no symptoms of CRS toxicity or neurotoxicity detected in tumor-bearing mice.

Blaeschke et al. (59) further explored the structural optimization of TIM-3/CD28 CSRs by engineering six variants with differing lengths of the TIM-3 and CD28 transmembrane domains. Their goal was to determine whether including larger parts of CD28 may enhance CSR function. This approach was informed by earlier findings from Oda et al. (62), who suggested that the inclusion of a cysteine bond in the CD28 extracellular domain promotes receptor multimerization, thereby strengthening CD28 signaling. Consistent with this, Blaeschke et al. (59) found that the two CSRs containing the largest CD28 domains induced the greatest levels of T cell proliferation and cytokine production. The study found that both generation anti-CD19 CARs expressing the CSR demonstrated higher CAR numbers, increased proliferative potential, increased CD25 expression, and decreased levels of late-effector phenotype. Interestingly, second-generation CAR T cells expressing the CSR had decreased percentages of cytokine-secreting cells, and interestingly, both generations expressing the CSR showed higher proliferative potential even in the absence of target cells, though reassuringly significant cytokine release was not detected in the absence of target cells. These unexpected findings raise important questions regarding the long-term implications of CSR-driven proliferation, including the potential for premature T cell exhaustion. Further *in vivo* studies are warranted to validate these results, evaluate durability and specificity, and assess safety profiles in preclinical models.

### 3.3 TIGIT-based stimulatory switch receptors

T cell immunoreceptor with Ig and ITIM domains (TIGIT) is a co-inhibitory immune checkpoint receptor that negatively regulates T cells and NK cells. TIGIT has a higher binding affinity than the stimulatory receptor CD226 for binding to CD155 and CD112 ligands (63). Upon binding to CD155, TIGIT inhibits T cell proliferation and activation. As with other immune checkpoint ligands such as PD-1 and CTLA-4, tumors diminish the immune response by overexpressing TIGIT ligands. To counter these inhibitory effects, studies have explored TIGIT-based CSRs (**Figure 3C**).

Hoogi et al. (63) developed two TIGIT/CD28 CSRs composed of the extracellular TIGIT domain, the intracellular CD28 domain, and the transmembrane domain of either TIGIT or CD28. They found that the CSR containing the TIGIT transmembrane domain demonstrated superior performance. When tested *in vitro* and in human tumor xenograft *in vivo*, the CSR enhanced cytokine secretion, delayed tumor growth, upregulated activation markers, and protected against T cell hypofunction following repeated antigen exposure when combined with a melanoma-specific TCR. The study further found that the CSR enhanced the functionality of anti-CD19 CARs in a manner dependent on CD155 expression by the target cells.

As CSRs have demonstrated significant potential in overcoming the challenges posed by the immunosuppressive TME, it has been of recent interest to investigate a CSR capable of simultaneously targeting multiple inhibitory checkpoint receptors. This may be particularly beneficial in cases when tumors develop mechanisms to circumvent the single checkpoint targeting strategies of most existing CSRs and in cases where CSRs targeting a single checkpoint pathway may not sufficiently achieve optimal anti-tumor effects. Additionally, such a strategy may further enhance the efficacy of CAR T cell therapy. Zhao et al. (64) were the first to develop a novel dual PD-1/TIGIT/CD28 CSR targeting both PD-1 ligand PD-L1 and TIGIT ligand CD155 by fusing the extracellular domains of PD-1 and TIGIT with the transmembrane and intracellular domain of CD28. Co-expression of the PD-1/TIGIT/CD28 CSR with an anti-EGFR CAR with the 4-1BB costimulatory domain resulted in enhanced cytokine release, proliferation and cytotoxicity *in vitro*. In xenograft mouse models, CSR-expressing CAR T cells reduced tumor progression and volume, increased overall survival, and rejected rechallenged tumors. Likewise, in patient-derived xenograft (PDX) mouse models, CSR-expressing CAR T cells demonstrated enhanced anti-tumor effects and robust infiltration. TIGIT-based CSRs show a great deal of promise but remain to be optimized before entering the clinical setting.

## 4 Targeting cytokines

**Figure 4:**
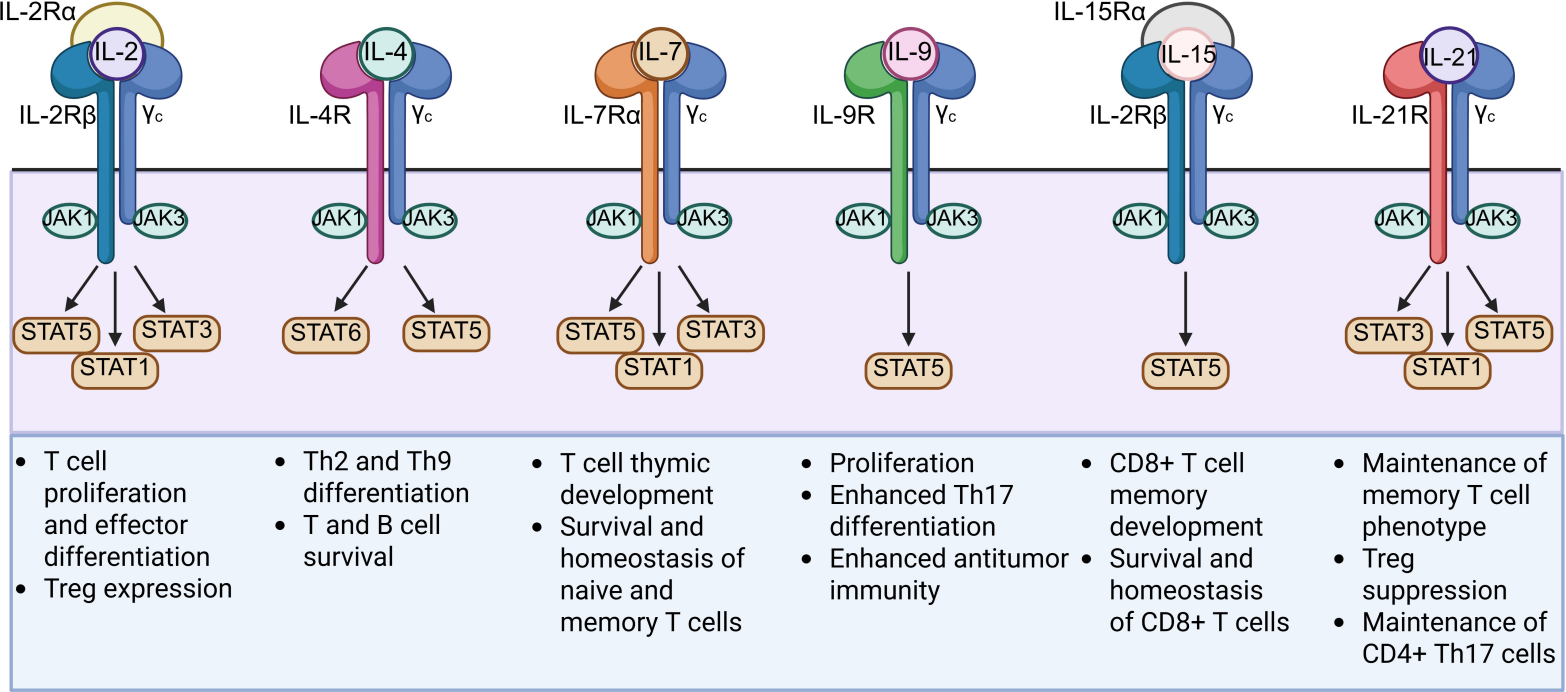
Structures and functions of the six members of the common γ chain (γc) cytokine family (IL-2, IL-4, IL-7, IL-9, IL-15, and IL-21) used for the intracellular domain of ICRs. γc cytokines play critical roles in T cell development and differentiation and primarily signal through the JAK-STAT pathway upon activation.

The TME consists of several immunosuppressive cytokines such as transforming growth factor-β (TGF-β), IL-4, and IL-10. These cytokines are capable of recruiting immunosuppressive cells such as myeloid-derived suppressor cells (MDSCs), Tregs, and tumor-associated macrophages (TAMs) in order to support tumor development and suppress CAR T cell antitumor responses (65). Cytokine signaling also plays a pivotal role in T cell function, proliferation, and differentiation (66). To enhance CAR T cell efficacy in the immunosuppressive TME, several chimeric receptors called inverted cytokine receptors (ICRs) have been explored (**Table 2**). These ICRs link the extracellular domain of an immunosuppressive cytokine receptor with the transmembrane and intracellular domains of an immunostimulatory cytokine receptor, converting an inhibitory signal into a stimulatory signal upon binding to the immunosuppressive cytokine. Recent studies developing ICRs have explored using the intracellular domain of cytokines belonging to the common γ chain (γc) cytokine family, which includes IL-2, IL-4, IL-7, IL-9, IL-15, and IL-21 (**Figure 4**) (66).

**Table 2:** Inverted cytokine receptors (ICRs) designed to supplement CAR T cells.

Cytokine category	ICR type	Extracellular domain	Intracellular domain	CAR target antigen	Cancer type	CAR/ICR efficacy	Reference
IL-4 Based	4/2	IL-4Rα	IL-2Rβ	MUC1, PSMA	Prostate	Enhanced CAR T cell cytolytic activity and proliferation via STAT3/STAT5/ERK phosphorylation in response to IL-4.	(67)
	4/7	IL-4Rα	IL-7Rα	PSCA, MUC1	Pancreatic, Breast	Improved T cell proliferation and antitumor activity in IL-4-rich environments; no significant increase in cytotoxicity observed.	(68, 69)
	4/15	IL-4Rα	IL-15Rβ	NKG2D	Pancreatic	Increased expansion, activation, cytotoxicity, and cytokine release; reduced exhaustion and higher proportion of less differentiated T cell phenotypes	(70)
	4/21	IL-4R	IL-21R	Not specified	Not specified	Superior antitumor efficacy; retained cytotoxicity and reduced exhaustion in the presence of IL-4, potentially due to activation of STAT3 and polarization to Th17-like phenotype	(71)
TGF-β-Based	TGF-β/IL-2/15	TGF-βR	IL-2/IL-15 Rβ	STEAP1	Prostate	Mitigated exhaustion; enhanced proliferation, cytokine secretion, and cytotoxicity of CAR T cells *in vitro*	(20)
	TGF-β/IL-7	TGF-βR	IL-7Rα	PSMA, CD19	Prostate, B-cell Lymphoma	Enhanced proliferation and cytotoxicity *in vitro*; improved antitumor effects and higher cytokine release *in vivo*	(18, 21)
Other	GM-CSF/IL-18	GM-CSFRα/β	IL-18Rα/β	Not specified	Solid Tumors	Increased expansion and cytokine production upon chronic antigen exposure; potent antitumor activity *in vivo* at lower cell doses	(22)
	IL-6/IL-7	IL-6Rα/β	IL-7Rα	CD19	Solid Tumors	Enhanced proliferation upon antigen stimulation; elevated STAT3 and STAT5 phosphorylation; effectively sequestered monocyte-derived IL-6 and IL-1β associated with CRS and neurotoxicity *in vitro*	(72)
	Multi CSR/ICR	TGF-βR, IL-4Rα	4-1BB, IL-7Rα	PSCA	Not specified	Enhanced expansion, cytokine secretion, and proliferation in the presence of PSCA, TGF-β, and IL-4; no significant differences in activation, exhaustion, and memory profile compared to unmodified CARs	(73)

ICR, Inverted Cytokine Receptor; CAR, Chimeric Antigen Receptor; MUC1, Mucin-1; PSMA, Prostate-specific membrane antigen; STAT3, Signal transducer and activator of transcription 3; STAT5, Signal transducer and activator of transcription 5; PSCA, Prostate stem cell antigen; NKG2D, Natural-killer group 2, member D; Th17, T helper 17; STEAP1, Six transmembrane epithelial antigen of the prostate 1 (STEAP1); GM-CSF, Granulocyte-macrophage colony-stimulating factor; CRS, cytokine release syndrome.

### 4.1 IL-4-targeting ICRs

Multiple cancers including renal cell carcinoma, melanoma, breast, glioblastoma, lung, prostate, bladder and head and neck cancers have been found to have increased IL-4 receptor (IL-4R) expression (66). Several ICRs have been developed to enhance CAR T cell functionality in the TME by sequestering immunosuppressive IL-4. These ICRs simultaneously limit IL-4 availability to tumors and convert immunosuppressive signals into stimulatory signals. One of the first groups to develop an ICR, Wilkie et al. (67) designed a 4αβ ICR by fusing the IL-4Rα extracellular domain with the transmembrane and intracellular domains of IL-2Rβ, which is shared by IL-2 and IL-15. Upon TCR and costimulatory signaling, activated T cells produce IL-2 to stimulate proliferation. Although IL-2 also drives effector differentiation of naive T cells, IL-2Rβ signaling has been found to enhance the CAR T cell efficacy. The study found that MUC1- or PSMA-CAR T cells expressing the 4αβ ICR exhibited increased cytolytic activity and exponential proliferation via STAT3/STAT5/ERK phosphorylation in response to IL-4.

### 4.2 IL-4/IL-7 ICR + CAR

IL-7 promotes the survival and homeostatic expansion of naive and memory T cells and has been used for *in vitro* expansion of CAR T cells (66). Mohammed et al. (68) composed an IL-4/IL-7 ICR by fusing the IL-4Rα ectodomain with the transmembrane and intracellular domains of IL-7Rα and investigated the *in vitro* and *in vivo* potential of prostate stem cell antigen-targeting CARs (CAR-PSCA) expressing the ICR in treating pancreatic cancer, which is characterized by high IL-4 levels and PSCA expression. The study found that the ICR-expressing T cells exhibited enhanced T cell proliferation, and CAR-PSCA cells expressing the ICR displayed enhanced antitumor activity in an IL-4 and PSCA-dependent manner. In a similar study, Bajgain et al. (69) explored the *in vitro* and *in vivo* potential of mucin 1-targeting CARs (MUC1-CAR) expressing an IL-4/IL-7 ICR in the IL-4 rich and PSCA-upregulated breast cancer microenvironment. ICR-expressing second-generation MUC1-CARs demonstrated enhanced antitumor activity and proliferation, while ICR-expressing first generation MUC1-CARs expanded but exhibited an exhausted phenotype and failed to produce superior antitumor effects. Interestingly, neither study noted increases in cytotoxicity with the addition of the ICR to the CAR.

### 4.3 IL-4/IL-15 ICR+CAR

IL-15 has been found to enhance CD8+ T cell and NK expansion and function (66). Zhou et al. (70) developed a novel natural killer group 2, member D (NKG2D)-targeting CAR construct expressing a IL-4/IL-15 ICR consisting of the IL-4R extracellular domain linked to the transmembrane and intracellular domains of IL-15. NKG2D receptors play a role in tumor immunosurveillance and are expressed on the surface of immune cells such as T cells and NK cells (74). Ligands for NKG2D (NKG2DL) are expressed on stressed cells. In the process of malignant transformation, tumor cells undergo various forms of stress, including DNA damage, activation of heat shock proteins, and oxidative stress (75). These forms of cellular stress induce the expression of NKG2D ligands (NKG2DL) on tumor cells. The study found that the CAR/ICR construct demonstrated increased expansion and activation, cytotoxicity, and cytokine release in the pancreatic cancer TME both *in vitro* and *in vivo*. Additionally, the CAR/ICR construct mitigated exhaustion and increased the proportion of less differentiated T cell phenotypes *in vitro*. However, while the NKG2D pathway is a viable avenue, tumors may deploy countermeasures including proteolytic-mediated shedding of NKG2DL or exosome-mediated secretion to release soluble NKG2D ligands in order to evade NKG2D surveillance (76, 77). Elevated levels of soluble NKG2D ligands are associated with worsened patient outcomes for several cancer types (75–77). These forms of immune evasion may pose challenges to the efficacy of the CAR.

### 4.4 IL-4/IL-21 ICR+CAR

IL-21 has been found to improve antitumor T cell immunity by preventing T cell differentiation and inhibiting Treg expansion (66). Wang et al. (71) designed an IL-4/IL-21 ICR that demonstrated superior anti-tumor efficacy *in vitro* and *in vivo* in combination with CARs in comparison to IL-4/IL-7 CAR/ICRs. Unlike IL-4/IL-7 CAR/ICRs, IL-4/IL-21 CAR/ICRs retained cytotoxicity and demonstrated attenuated exhaustion in the presence of immunosuppressive IL-4. This may in part be explained by the differing phospho-STAT signaling cascades activated by each ICR. The IL-4/IL-7 ICR activates STAT5 phosphorylation and promotes Th1 differentiation, while the IL-4/IL-21 ICR activates STAT3 phosphorylation and polarization to the Th17-like phenotype, which exhibit lower exhaustion markers than Th1 cells. As IL-21 is a pleiotropic cytokine and may be influenced by the presence of other cytokines, further studies are needed to investigate safety and efficacy when the ICR constructs are clinically translated.

### 4.5 TGF-β-targeting ICRs

TGF-β plays a key paradoxical role in cancer progression and is involved in regulating various cancer cell functions such as cell cycle progression, apoptosis, and differentiation (78). Its effects on cancer progression can vary with tumor type and genetic landscape. Interestingly, it switches from tumor suppressive in early-stage tumors to tumor promoter in later-stage tumors (79). In normal and premalignant cells, TGF-β primarily functions as a tumor suppressor by inhibiting cell proliferation and inducing apoptosis (80). However, tumor cells can selectively bypass TGF-β-mediated growth inhibition by activating oncogenes via mutations and inactivating mutations in tumor suppressor genes (80). By inhibiting TGF-β-mediated growth inhibition, tumor cells then take advantage of TGF-β signaling to increase the epithelial-to-mesenchymal transition (EMT), promoting their own migration and invasion abilities (80). TGF-β can also act in a paracrine manner and shape the TME by activating cancer-associated fibroblasts (CAFs), promoting angiogenesis, and stimulating extracellular matrix production to promote cancer progression (80).

Similar to IL-4-based ICRs, several TGF-β-based ICRs have been developed by fusing the exodomain of the immunosuppressive TGF-β receptor to the transmembrane and intracellular domains of stimulatory receptors. Beck et al. (20) generated a TGF-β-IL-2Rβ CSR and found it mitigated exhaustion and enhanced the *in vitro* proliferation, cytokine secretion, and cytotoxicity of CAR T cells targeting STEAP1, a highly expressed protein in prostate cancer. Further studies are needed to determine the *in vivo* efficacy of the CSR as well as to identify potential off-target effects of the CAR/ICR T cells exhibiting enhanced activity in any environment outside the TME with high TGF-β concentrations. Weimin et al. (21) developed a TGF-β/IL-7 CSR co-expressed with CAR T cell targeting prostate-specific membrane antigen (PSMA) and found the CAR/ICR exhibited enhanced proliferation and cytotoxicity *in vitro* following repeated antigen activation by tumor cells. In mouse xenograft models, higher cytotoxicity and enhanced anti-tumor effects were noted with the CAR/ICR combination. Elevated cytokine release was also found in mouse PDX models. Noh et al. (18) found that anti-CD19 CAR T cells expressing a TGF-β/IL-7 CSR exhibited superior anti-tumor efficacy, including prolonged overall survival rates and the prevention of tumor recurrence in a murine model of CD19+ B cell lymphoma. While TGF-β-based ICRs in CAR-T therapy are promising and some early-stage clinical trials are exploring them, they have not yet been widely adopted in clinical settings.

### 4.6 Other ICRs

Several ICRs containing other cytokines have recently been developed to enhance the efficacy and specificity of CAR T cell therapy, particularly in the immunosuppressive TME of solid tumors. Repeated antigen stimulation in the TME is a major driver of CAR T cells cell dysfunction, leading to exhaustion and impaired persistence. Studies have found that an inducible costimulatory molecule capable of activating MyD88, the central signaling hub for Toll-like receptors and the IL-1 and IL-18 receptors, may help sustain CAR T cell effector functions when facing chronic antigen exposure in the TME (22). Lange et al. (22) identified the cytokine GM-CSF as a potential candidate due to its invariable expression after T cell activation. They developed a novel GM-CSF/IL-18 CSR that enhances the effector functions of CARs in an antigen- and activation-dependent manner by combining the extracellular domains of the α/β chains of GM-CSF receptor and transmembrane and intracellular domains of the α/β chains of the IL-18 receptor. This establishes an autocrine loop linking T cell activation, indicated by the expression of GM-CSF, with the MyD88 signaling pathway. The CAR/CSR exhibited greater expansion and cytokine production in repeat stimulation assays mimicking chronic antigen exposure as well as potent antitumor activity *in vivo* solid tumor xenograft models at lower cell doses than standard CAR T cells.

CRS remains a major limitation of CAR T cell therapy, with cytokine IL-6 being one of the most elevated cytokines during CRS episodes. To mitigate this toxicity, Yoshikawa et al. (72) designed a G6/7R CSR from the extracellular IL-6 domain and the transmembrane and intracellular IL-7 receptor that constitutively activates the JAK-STAT pathway important for various aspects of the immune response. *In vitro*, CSR-expressing anti-CD19 CAR T cells exhibited enhanced proliferation upon antigen stimulation, elevated phosphorylation of STAT3 and STAT5, and the ability to effectively sequester monocyte-derived IL-6 and IL-1β, which are associated with CRS and neurotoxicity, respectively. However, further studies are necessary to validate these *in vitro* findings and evaluate the relationship between CAR T cell dose, expansion, and serum IL-6 levels *in vivo*.

Recent studies have also explored strategies to increase the specificity of later generation CARs that target antigens such as CD19 which may be expressed on normal cells, potentially resulting in undesirable “on-target, off-tumor” toxic effects. Though rare, some patients treated with anti-CD19 CARs develop lifelong B-cell aplasia, as the target CD19 antigens are also expressed on non-malignant B cells. Similarly, anti-HER2 CAR T cells have been attributed to lethal toxic effects and CRS due to the expression of HER2 on normal tissues of vital organs. Sukumaran et al. (73) designed a novel study to test whether co-expressing an anti-PSCA first-generation CAR with both TGF-β/4-1BB CSR and 4/7 ICR could enhance CAR specificity at the tumor site. CARs expressing the CSR+ICR demonstrated enhanced expansion in the presence of PSCA, TGF-β and IL-4 while unmodified CARs failed to expand. CSR+ICR-expressing CARs also exhibited higher cytokine secretion and enhanced expansion. However, despite enhanced proliferation and cytokine production, there were no significant differences in activation markers, exhaustion phenotype, and memory subset distribution between modified and unmodified CAR T cells, highlighting the need for further refinement to improve selective activity and long-term safety.

## 5 Conclusion

CSRs offer a promising approach to improve the therapeutic efficacy of CAR T cell therapies for solid tumors. The inhibitory-to-stimulatory design of CSRs involving key checkpoint inhibitors holds great potential in mitigating challenges to CAR T cell therapy presented by the immunosuppressive TME of solid tumors such as T cell exhaustion and decreased cytotoxicity. In designing these receptors, safety remains a critical consideration, given that some studies have found potential side effects such as autoimmune reactions and cytokine release syndrome. Additional *in vitro* and *in vivo* studies are thus critical to investigate unexpected immune interactions, assess long-term safety, and identify potential off-target effects before advancing to clinical trials.

Looking ahead, expanding CSR designs to target a broader array of inhibitory receptors and combining them rationally with ICRs offers an exciting avenue to further improve tumor specificity and reduce toxicity. Furthermore, emerging synthetic receptor platforms, such as synthetic Notch (synNotch) receptors (81–86), enable logic-gated antigen sensing and inducible expression of therapeutic payloads, including CSRs or cytokines. These systems allow for context-specific activation of T cells within the TME, offering an additional layer of control to enhance precision and minimize off-tumor effects. Such integrated and programmable strategies hold great promise for refining the precision and therapeutic index of next-generation engineered T cell therapies.
